# Supplementary material for: Fertilization using manure minimizes the trade-offs between biodiversity and forage production in agri-environment scheme grasslands
Source: PLoS One. 2023 Oct 4;18(10):e0290843. doi: 10.1371/journal.pone.0290843 (PMC10550152; doi:10.1371/journal.pone.0290843)
Supplement: S1 File — (PDF) [file pone.0290843.s002.pdf]

Supplementary information

Fertilization by manure minimizes the trade-offs between biodiversity and forage production in agri-environment scheme grasslands

Contents

METHODS 2

Section 1 Colt Park trail experiment 2

Section 2 Estimation of livestock production 2

Fig 1. 4

Fig 2. 3

RESULTS 5

Table 1. 5

Table 2. 6

Table 3. 7

Table 4. 8

Table 5 9

Table 6 10

REFERENCES 12

METHODS

Section 1 Colt Park trail experiment

Colt Park trail is a long-term nutrient and plant biodiversity manipulation experiment located at 300 m altitude in the Ingleborough National Nature Reserve in North Yorkshire, England (54°12'N, 2°21'W). The field trial started in 1990 on permanent grassland dominated by the perennial grass species *Lolium perenne* and *Cynosurus cristatus*, on a shallow brown-earth soil (pH 5.1) over limestone of moderate-high residual fertility. The aim of the experiment was to test different management strategies for improving the diversity of grasslands in a working agricultural context (1, 2).

The experiment consists of 72 plots, each 2.5 m x 6 m (15 m2) in size, arranged in three blocks of 24 plots. Each of the three blocks is subdivided into three sub-blocks of 8 plots corresponding to three sowing treatments applied during 2004-2008 with no remnant effect on the biological communities studied (3). For this, experiment we used only the plots under no seed addition treatment, this selection results in 8 plots per block for a total of 24 plots. In each block three fertilizer treatments have been applied to two plots randomly chosen plots: N:P: K fertilizer (20:10:10, 25 kg ha^{-1} nitrogen plus 12.5 kg ha^{-1} of P2O2 and K2O; hereafter NPK,), farmyard manure (12 t ha^{-1} hereafter FYM), N:P: K fertilizer + farmyard manure (hereafter NPK+FYM) and a further two plots are left as a control with no fertilizer. In total, there are 6 plots per treatment. The quantities are according the limits of the environmental stewardship, of 12,500 kg (total rate of nitrogen must not exceed the 100 kg/ha), per hectare per year for FYM and 50kg for inorganic fertilizer (4) versus 91 kg/ha of inorganic fertilizer outside stewardship agreements, rising to well over 200 kg for multiple harvest silage systems (5).

Section 2 Estimation of livestock production

To estimate the number of animals that could be fed with the hay produced under each treatment, we first estimated the amount of hay that would be available for daily animal consumption under each treatment. To this end we divided the amount of hay that would be available for animal consumption everyday by the amount of hay that an animal would need to consume daily to reach its nutritional needs. To this end, we first transformed the mean grams per meter of hay produced and scaled up to one hectare. Then, we calculated the amount of hay that could be consumed in a day by the cattle. This was done to ensure that the hay produced lasted the standard 24-week period that the cattle are reared indoors during the winter (16). The latter was estimated by

dividing the amount of hay produced in one hectare by the total number of days the cattle are indoors (168 days). Finally, we divided the amount of hay by the amount an animal would need to consume considering the metabolizable energy of the hay using the app FarmIQ (2016).

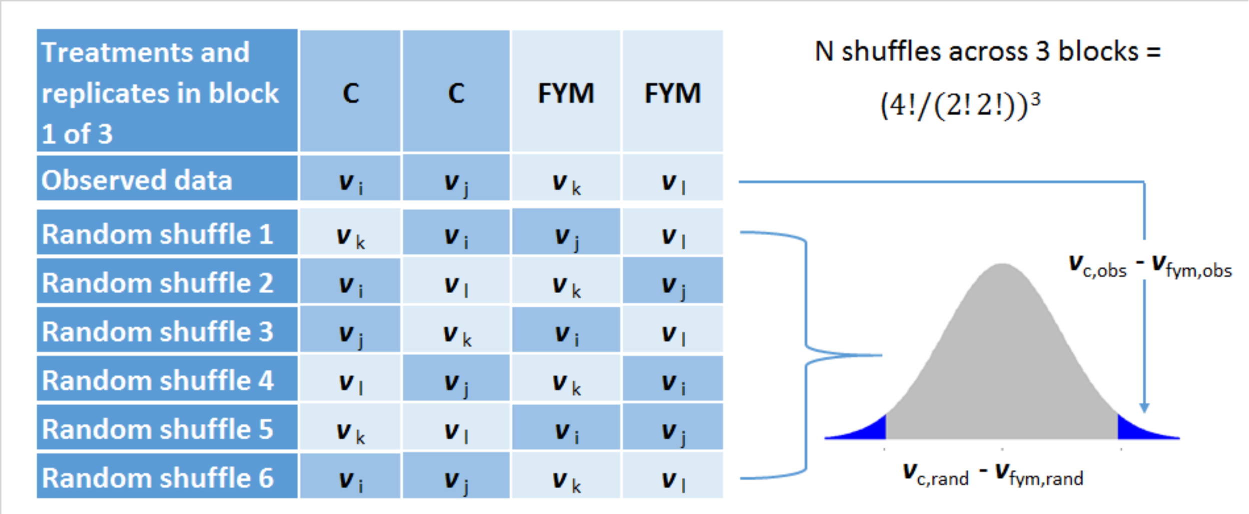

Fig 1. The randomization testing scheme is shown for one of the three experimental blocks and for one pair of treatments only. In each cell **v** denotes the vector of ranked measurements across the 12 indicators min-max normalized. Two test statistics were generated for the observed vectors: the difference in weighted mean and weighted standard deviation of the indicators in each vector between each pair of treatments. The same test statistics were calculated for each random reshuffle of the data. Hence vectors were swapped around within each block but with the randomization carried out across all blocks simultaneously. Test statistics were calculated for each randomization, and these form a reference distribution. A P value conditional on the reference distribution was then calculated as the proportion of times the absolute value of each observed test statistic was greater or equal to each randomized test statistic. Significant P values would fall in the tails of the reference distribution leading us to reject the null hypothesis of no difference between the observed and randomized test statistics for the pair of treatments.

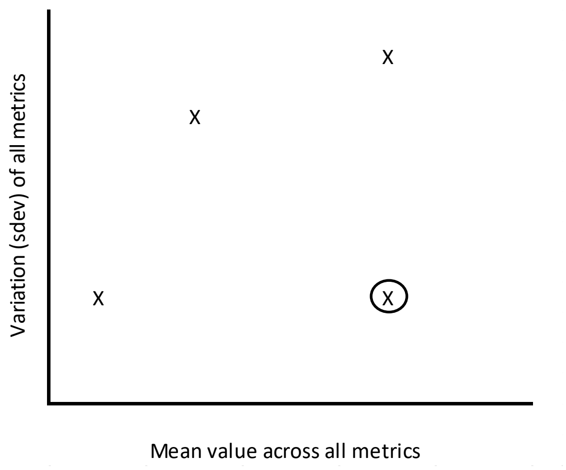

Fig 2. Optimizing values across variables for each treatment. The x-axis represents the mean across variables while y-axis represents the variation or standard deviation around the mean. Due to unequal number of variables, we compared the weighted mean and the weighted standard deviation. We defined as optimization as the point with the highest weighted mean but the lowest weighted standard deviation. In this sense the optimal treatment is that with the highest weighted mean and lowest variation around the mean (measured as the weighted standard deviation), represented with a circle in the figure.

RESULTS

Table 1. Results of the Pearson correlation test between variables. Forage Re: Ruminant metabolizable energy. Mw: Mean dry weight 2011-2014. Cp: Crude protein. As: Ash. Insect community: Pp: Percent of parasitism. Dpo: Shannon-Weiner diversity of pollinators. Dpa: Shannon-Weiner diversity of parasitoids. Dh: Shannon-Weiner diversity of herbivores. Ba: Abundance of bumblebees. Plant community: Np: nectar productivity. Fs: Forb species richness. Dpl: Diversity of plants. N=24**.**

	**Re**	**Cp**	**As**	**Mw**	**Pp**	**Ba**	**Dpol**	**Dh**	**Dpa**	**Dpl**	**Fs**	**Np**
**Re**	1	0.48	-0.26	0.17	0.31	0.04	-0.08	0.18	-0.01	0.26	-0.16	-0.08
**Cp**	0.48	1	-0.29	0.13	0.09	-0.38	-0.39	-0.04	-0.06	-0.15	-0.36	-0.35
**As**	-0.26	-0.29	1	0.19	0.38	-0.08	-0.11	-0.15	-0.14	-0.15	-0.16	-0.27
**Mw**	0.17	0.13	0.19	1	0.54	-0.29	-0.17	0.04	0.21	-0.05	-0.45	-0.36
**Pp**	0.31	0.09	0.38	0.54	1	-0.11	-0.35	0.04	0.19	-0.05	-0.22	-0.23
**Ba**	0,03	-0,42	-0,06	-0,28	-0,17	1	0,35	0,09	-0,12	0,09	0,55	0,66
**Dpol**	-0.08	-0.39	-0.11	-0.17	-0.35	0.31	1	0.18	0.18	0.16	0.29	0.44
**Dh**	0.18	-0.04	-0.15	0.04	0.04	0.09	0.18	1	0.74	0.43	-0.38	-0.03
**Dpa**	-0.01	-0.06	-0.14	0.21	0.19	-0.14	0.18	0.74	1	0.18	-0.41	-0.07
**Dpl**	0.26	-0.15	-0.15	-0.05	-0.05	0.06	0.16	0.43	0.18	1	0.14	0.09
**Fs**	-0,16	-0,36	-0,16	-0,45	-0,22	0,55	0,29	-0,38	-0,41	0,14	1	0,77
**Np**	-0.08	-0.35	-0.27	-0.36	-0.23	0.69	0.44	-0.03	-0.07	0.09	0.77	1

Table 2. Performance of each variable for each fertilizer treatment. Values were min-max normalized so values can be compared between treatments and across variables. The mean value for each group of variables is reported as well as the weighted mean across variables. We weighted the value by considering that variables of forage should count for the 50% of the mean value and variables insect community and plant community will contribute for the other 50%. Neutral cellulase gammanase digestibility was removed from the analysis due to high correlation with ruminant metabolizable energy. Standard errors are reported next to mean values.

Variable	No-fertilizer	FYM	NPK	NPK+FYM
**Forage**				
Ash	0.61± 0.05	0.55 ± 0.1	0.55 ± 0.1	0.59 ± 0.14
Crude protein	0.14 ± 0.06	0.54 ± 0.1	0.34 ± 0.1	0.55 ± 0.1
Ruminant metabolizable energy	0.64 ± 0.04	0.81 ±0.05	0.51 ± 0.1	0.71 ± 0.1
Mean dry content 2011-2014	0.26 ± 0.06	0.60 ±0.03	0.51 ±0.04	0.66 ± 0.14
**Mean forage**	**0.41 ± 0.13**	**0.62 ± 0.06**	**0.48 ± 0.05**	**0.63 ± 0.04**
**Insect community**				
Abundance of *Bombus* spp.	0.38 ± 0.15	0.17 ± 0.12	0.12 ±0.05	0.00 ± 0
Diversity of herbivores	0.46 ± 0.06	0.48 ± 0.1	0.33 ± 0.1	0.50 ± 0.14
Diversity of parasitoids	0.37 ± 0.06	0.32 ± 0.1	0.36 ± 0.1	0.54 ± 0.14
Diversity of pollinators	0.68 ± 0.1	0.58 ±0.07	0.68 ± 0.1	0.53 ± 0.13
Percent parasitism	0.47 ± 0.04	0.71 ± 0.1	0.38 ± 0.1	0.62 ± 0.15
**Mean insect community**	**0.47 ±0.06**	**0.45 ± 0.1**	**0.38 ± 0.1**	**0.44 ± 0.1**
**Plant community**				
Diversity of plants	0.52 ± 0.11	0.35 ± 0.1	0.47 ±0.12	0.26 ± 0.15
Forb species richness	0.75 ± 0.1	0.56 ± 0.1	0.69 ±0.1	0.17 ± 0.04
Nectar productivity	0.47 ± 0.13	0.27 ± 0.1	0.30 ±0.1	0.03 ± 0.02
**Mean plant community**	**0.58 ± 0.08**	**0.40 ± 0.1**	**0.49 ± 0.1**	**0.15 ± 0.07**
**Weighted mean across variables**	**0.47 ± 0.05**	**0.52 ± 0.05**	**0.46 ± 0.04**	**0.46 ± 0.07**

Table 3. Probability that first treatment (T1) is higher than the second treatment (T2; P(T1 >T2)) and probability that T1 is lower than T2 (P(T1< T2)) for each of the pair comparisons for the mean across variables within each group. The p values correspond to the of times out of 10,000 randomizations the mean of the treatment T1 was lower/higher than the mean of the treatment T2. SIG stands for significant (P <0.05).

Group of variables	Treatments	P (T1 >T2)	P (T1< T2)	SIG
Forage	No-fertilizer_FYM	0.0041	0.9959	*
	No-fertilizer_NPK	0.1034	0.8966	
	No-fertilizer_NPK+FYM	0.0047	0.9953	*
	FYM_NPK	0.9668	0.0332	*
	FYM_NPK+FYM	0.4783	0.5217	
	NPK_NPK+FYM	0.0248	0.9752	*
Insect community	No-fertilizer_FYM	0.605	0.395	
	No-fertilizer_NPK	0.8991	0.1009	
	No-fertilizer_NPK+FYM	0.6623	0.3377	
	FYM_NPK	0.806	0.194	
	FYM_NPK+FYM	0.598	0.402	
	NPK_NPK+FYM	0.2749	0.7251	
Plant community	No-fertilizer_FYM	0.9905	0.0095	*
	No-fertilizer_NPK	0.8657	0.1343	
	No-fertilizer_NPK+FYM	0.9953	0.0047	*
	FYM_NPK	0.1675	0.8325	
	FYM_NPK+FYM	0.9684	0.0316	*
	NPK_NPK+FYM	0.9913	0.0087	*

Table 4. The probability of each treatment of maximizing the mean across variables and minimizing the variance around the mean. The probability value corresponds to the number of times out of the 10,000 randomizations the treatment on the left has a higher value than the treatment on the top. We considered probabilities values to be significant lower when P = < 0.05 and significant higher when P => 0.95. Those significant values are indicated with *.

	No-fertilizer	FYM	NPK	NPK+FYM
**Weighted mean across variables**				
**No-fertilizer**	NA	0.0297*	0.6128	0.5742
**FYM**		NA	0.9694*	0.8973
**NPK**			NA	0.4757
**NPK +FYM**				NA
**Weighted standard deviation across variables**				
**No-fertilizer**	NA	0.2584	0.5729	0.004*
**FYM**		NA	0.7126	0.0084*
**NPK**			NA	0.0115*
**NPK +FYM**				NA

Table 5. Livestock production under fertilizer treatment. Forage production and feeding value relative to winter requirements of spring-calving suckler cows and April-lambing mixed age ewes.

Variable	No-fertilizer	FYM	NPK	NPK+FYM
Suckler cows (600 kg)				
Mean number of cows per hectare	2.67 ± 0.3	3.83 ± 0.2	3.33 ± 0.2	4 ± 0.4
Metabolizable energy (MJ/kg)	8.82 ± 0.11	*9.31* ± 0.15	*8.4* ± 0.35	*9.03* ± 0.25
Mixed aged ewes				
Mean number of ewes per hectare	17.3 ± 1.3	24.7 ± 0.9	22.8 ± 0.8	25.8 ± 3.2
Metabolizable ruminant energy (MJ/kg)	8.82 ± 0.11	*9.31* ± 0.1	*8.4* ± 0.35	*9.03* ± 0.25

Table 6. List of species.

Group	Order	Family	Species
Herbivore	Diptera	AGROMYZIDAE	*Agromyza nana* Meigen, 1830
Herbivore	Diptera	AGROMYZIDAE	*Agromyza nigripes* Meigen, 1830
Herbivore	Diptera	AGROMYZIDAE	*Cerodonta denticorni*s (Panzer, [1806])
Herbivore	Diptera	AGROMYZIDAE	*Cerodontha fulvipes* (Meigen, 1830)
Herbivore	Diptera	AGROMYZIDAE	*Chromatomyia milii* (Kaltenbach, 1864)
Herbivore	Diptera	AGROMYZIDAE	*Chromatomyia nigra* (Meigen, 1830)
Herbivore	Diptera	AGROMYZIDAE	*Lyriomyza flaveol*a (Fallén, 1823a)
Herbivore	Diptera	AGROMYZIDAE	*Phytomyza crassiseta* Zetterstedt, 1860
Herbivore	Diptera	AGROMYZIDAE	*Phytomyza fallaciosa* Brischke, 1880
Herbivore	Diptera	AGROMYZIDAE	*Phytomyza leucanthemi* Hering, 1935
Herbivore	Diptera	AGROMYZIDAE	*Phytomyza notata* Meigen, 1830
Herbivore	Diptera	AGROMYZIDAE	*Phytomyza plantaginis* Goureau, 1851
Herbivore	Diptera	AGROMYZIDAE	*Phytomyza ranunculi* (Schrank, 1803)
Herbivore	Diptera	AGROMYZIDAE	*Phytomyza ranunculivora* Hering, 1932
Herbivore	Diptera	AGROMYZIDAE	*Phytomyza stolonifera* Hering, 1949
Herbivore	Diptera	ANTHOMYIIDAE	*Pegomyia solennis* (Meigen, 1826)
Herbivore	Diptera	DROSOPHILIDAE	*Scaptomyza flava* (Fallén, 1823)
Herbivore	Diptera	DROSOPHILIDAE	*Scaptomyza graminum* (Fallén, 1823)
Herbivore	Diptera	EPHYDRIDAE	*Hydrellia maura* Meigen, 1838
Herbivore	Diptera		Diptera sp.1
Herbivore	Diptera		Diptera sp.2
Herbivore	Diptera		Diptera sp.3
Herbivore	Diptera		Diptera sp.4
Herbivore	Diptera		Diptera sp.5
Herbivore	Lepidoptera	ELASCHISTIDAE	*Elaschita* sp.
Parasitoid	Hymenoptera	BRACONIDAE	*Apodesmia irregularis* (Wesmael, 1835)
Parasitoid	Hymenoptera	BRACONIDAE	*Apodesmia similis* (Szépligeti, 1898)
Parasitoid	Hymenoptera	BRACONIDAE	*Chorebus aphantus* (Marshall, 1896)
Parasitoid	Hymenoptera	BRACONIDAE	*Chorebus asramenes* (Nixon, 1945)
Parasitoid	Hymenoptera	BRACONIDAE	*Chorebus conjugens* (Nees von Esenbeck, 1812)
Parasitoid	Hymenoptera	BRACONIDAE	*Chorebus longicornis* (Nees, 1811)
Parasitoid	Hymenoptera	BRACONIDAE	*Dacnusa areolaris* (Nees, 1811)
Parasitoid	Hymenoptera	BRACONIDAE	*Dacnusa faeroeensis (Roman, 1917)*
Parasitoid	Hymenoptera	BRACONIDAE	*Dacnusa laevipectis* Thomson, 1895
Parasitoid	Hymenoptera	BRACONIDAE	*Dacnusa macrospila* (Haliday, 1839)
Parasitoid	Hymenoptera	BRACONIDAE	*Dacnusa maculipes* Thomson, 1895
Parasitoid	Hymenoptera	BRACONIDAE	*Dacnusa plantaginis* Griffiths, 1967
Parasitoid	Hymenoptera	BRACONIDAE	*Exotela gilvipes* (Haliday, 1839)
Parasitoid	Hymenoptera	BRACONIDAE	*Grammospila rufiventris* (Nees, 1812)
Parasitoid	Hymenoptera	BRACONIDAE	*Phaedrotoma caesa* (Haliday, 1837)
Parasitoid	Hymenoptera	BRACONIDAE	*Phaedrotoma* sp.BOLD:ADF0710
Parasitoid	Hymenoptera	EULOPHIDAE	*Chrysocharis viridis* (Nees, 1834)
Parasitoid	Hymenoptera	EULOPHIDAE	*Diglyphus isaea* (Walker)
Parasitoid	Hymenoptera	EULOPHIDAE	*Hemiptarsenus unguicellus* Westwood, 1833
Parasitoid	Hymenoptera	EULOPHIDAE	*Pnigalio* sp.BOLD:ADI0573
Parasitoid	Hymenoptera	PLATYGASTRIDAE	Platygastridae sp.BOLD:ADH7962
Parasitoid	Hymenoptera	PTEROMALIDAE	*Microgaster maculata* Walker, 1833
Pollinator	Coleoptera	CANTHARIDAE	*Rhagonycha fulva* (Scopoli, 1763)
Pollinator	Coleoptera	DASCILLIDAE	*Dascillus ceruinus* (Linnaeus, 1758)
Pollinator	Coleoptera	NITIDULIDAE	*Meligethes* aff. *aeneus* (Fabricius, 1775)
Pollinator	Diptera	ANTHOMYIIDAE	*Botanophila* sp.
Pollinator	Diptera	ANTHOMYIIDAE	*Botanophila striolata* (Fallén, 1824)
Pollinator	Diptera	ANTHOMYIIDAE	*Delia platura* (Meigen, 1826)

Group	Order	Family	Species
Pollinator	Diptera	ANTHOMYIIDAE	*Delia* sp.
Pollinator	Diptera	ANTHOMYIIDAE	*Hylemya variata* (Fallén, 1823)
Pollinator	Diptera	ANTHOMYIIDAE	*Pegoplata aestiva* (Meigen, 1826)
Pollinator	Diptera	ANTHOMYIIDAE	*Pegoplata infirma* (Meigen, 1826)
Pollinator	Diptera	CALLIPHORIDAE	*Lucilia caesar* (Linnaeus, 1758)
Pollinator	Diptera	CALLIPHORIDAE	*Melinda cognata* (Meigen, 1826)
Pollinator	Diptera	CERATOPOGONIDAE	*Mallochohelea* sp.
Pollinator	Diptera	DOLICHOPODIDAE	*Dolichopus plumipes* (Scopoli, 1763)
Pollinator	Diptera	DOLICHOPODIDAE	*Thyripticus* sp.
Pollinator	Diptera	EMPIDIDAE	*Empis livida* Linnaeus, 1758
Pollinator	Diptera	EMPIDIDAE	*Empis punctata* Meigen, 1804
Pollinator	Diptera	EPHYDRIDAE	*Hydrellia maura* Meigen, 1838
Pollinator	Diptera	EPHYDRIDAE	*Hydrellia* sp.
Pollinator	Diptera	FANNIIDAE	*Fannia* sp.
Pollinator	Diptera	FANNIIDAE	*Fannia umbratica* Collin, 1939
Pollinator	Diptera	MUSCIDAE	*Drymeia brumalis* (Rondani, 1866)
Pollinator	Diptera	MUSCIDAE	*Drymeia hastata* (Harris, [1780])
Pollinator	Diptera	MUSCIDAE	*Helina* sp.
Pollinator	Diptera	MUSCIDAE	*Hydrotaea irritans* (Fallén, 1823)
Pollinator	Diptera	MUSCIDAE	*Phaonia incana* (Wiedemann, 1817)
Pollinator	Diptera	MUSCIDAE	*Phaonia serva* (Meigen, 1826)
Pollinator	Diptera	MUSCIDAE	Thricops *sp.*
Pollinator	Diptera	OPOMYZIDAE	*Geomyza tripunctata* Fallén, 1823
Pollinator	Diptera	OPOMYZIDAE	*Opomyza germinationis* (Linnaeus, 1758)
Pollinator	Diptera	OPOMYZIDAE	*Opomyza petrei* Mesnil, 1934
Pollinator	Diptera	SCATHOPHAGIDAE	*Scathophaga stercoraria* (Linnaeus, 1758)
Pollinator	Diptera	SCATHOPHAGIDAE	Scathophagidae sp.
Pollinator	Diptera	SEPSIDAE	*Themira lucida* (Staeger in Schiødte, 1844)
Pollinator	Diptera	SYRPHIDAE	*Cheilosia albitarsis/ranunculi*
Pollinator	Diptera	SYRPHIDAE	*Chrysogaster virescens* Loew, 1854
Pollinator	Diptera	SYRPHIDAE	*Episyrphus balteatus* (DeGeer)
Pollinator	Diptera	SYRPHIDAE	*Eristalis horticola* (De Geer, 1776)
Pollinator	Diptera	SYRPHIDAE	*Eristalis tenax* (Linnaeus, 1758)
Pollinator	Diptera	SYRPHIDAE	*Eupeodes corollae* (Fabricius, 1794)
Pollinator	Diptera	SYRPHIDAE	*Lejogaster metallina* (Fabricius, 1781)
Pollinator	Diptera	SYRPHIDAE	*Melanostoma mellinum* (Linnaeus, 1758)
Pollinator	Diptera	SYRPHIDAE	*Platycheirus albimanus* (Fabricius, 1781)
Pollinator	Diptera	SYRPHIDAE	*Platycheirus manicatus* (Meigen, 1822)
Pollinator	Diptera	SYRPHIDAE	*Platycheirus rosarum* (Fabricius, 1787)
Pollinator	Diptera	SYRPHIDAE	*Rhingia campestris* (Meigen, 1822)
Pollinator	Diptera	SYRPHIDAE	*Sericomyia silentis* (Harris, 1778)
Pollinator	Diptera	SYRPHIDAE	*Sphaerophoria fatarum* Goeldlin, 1989
Pollinator	Diptera	SYRPHIDAE	*Sphaerophoria* sp.
Pollinator	Diptera	SYRPHIDAE	*Syritta pipiens* (Linnaeus, 1758)
Pollinator	Diptera	SYRPHIDAE	*Syrphus vitripennis* Meigen, 1822
Pollinator	Diptera	TABANIDAE	*Haematopota pluvialis* (Linnaeus, 1758)
Pollinator	Diptera	TACHINIDAE	Tachinidae sp.
Pollinator	Diptera	THEREVIDAE	*Dialineura anilis* (Linnaeus, 1761)
Pollinator	Heteroptera	CICADELLIDAE	Cicadellidae sp.
Pollinator	Heteroptera	MIRIDAE	Miridae sp.
Pollinator	Hymenoptera	APIDAE	*Bombus hortorum* (Linnaeus, 1761)
Pollinator	Hymenoptera	APIDAE	*Bombus lucorum* (Linnaeus, 1761)
Pollinator	Hymenoptera	APIDAE	*Bombus pascuorum* (Scopoli, 1763)
Pollinator	Hymenoptera	APIDAE	*Bombus terrestris* (Linnaeus, 1758)
Pollinator	Hymenoptera	ICHNEUMONIDAE	Ichneumonidae sp.1
Pollinator	Lepidoptera	CRAMBRIDAE	*Agriphila tristella* ([Denis & Schiffermuller], 1775)

REFERENCES

1. Smith RS (2005) *Ecological mechanisms affecting the restoration of diversity in agriculturally improved meadow grassland* (Defra Project BD1439) Available at: http://randd.defra.gov.uk/Default.aspx?Menu=Menu&Module=More&Location=None&Completed=0&ProjectID=9117.
2. Smith RS, et al. (2008) Long-term change in vegetation and soil microbial communities during the phased restoration of traditional meadow grassland. *J Appl Ecol* 45(2):670–679.
3. Villa-Galaviz E, Smart SM, Clare EL, Ward SE, Memmott J (2021) Differential effects of fertilizers on pollination and parasitoid interaction networks. *J Anim Ecol* 90(2):404–414.
4. Natural England (2013) *Entry Level Stewardship, Environmental Stewarhip Handbook*. Fourth.
5. AHDB (2019) *Nutrient Management Guide (RB209)* Available at: https://ahdb.org.uk/nutrient-management-guide-rb209.
6. Magurran AE (2004) *Measuring biological diversity* (Malden, Mass. Oxford : Blackwell, Oxford).
7. Allinson J, Natural England (2014) *Botanical surveys of Colt Park Meadows experimental plots 2010-2014, Ingleborough National Nature Reserve*.
8. Baude M, et al. (2016) Historical nectar assessment reveals the fall and rise of floral resources in Britain. *Nature* 530(7588):85–88.
9. Ivanova N V, Dewaard JR, Hebert PDN (2006) An inexpensive, automation-friendly protocol for recovering high-quality DNA. *Mol Ecol Resour* 6(4):998–1002.
10. Smith MA, Poyarkov NA, Hebert PDN (2008) DNA BARCODING: CO1 DNA barcoding amphibians: take the chance, meet the challenge. *Mol Ecol Notes* 8(2):235–246.
11. Hebert PDN, Cywinska A, Ball SL, DeWaard JR (2003) Biological identifications through DNA barcodes. *Proc R Soc B Biol Sci* 270(1512):313–321.
12. Ratnasingham S, Hebert PDN (2007) bold: The Barcode of Life Data System (http://www.barcodinglife.org). *Mol Ecol Notes* 7(3):355–364.
13. Oksanen J, et al. (2019) vegan: Community Ecology Package. R package version 2.5-4. Available at: https://cran.r-project.org/package=vegan.
14. Ward SE, Wilby A, Bardgett R (2016) *Managing grassland diversity for multiple ecosystem services* (Lancaster) Available at: http://sciencesearch.defra.gov.uk/Default.aspx?Menu=Menu&Module=More&Location=None&Completed=0&ProjectID=17251.
15. RDPE Northwest livestock Programme (2012) Analysing forage for winter feeding. 2018(24/04/2018). Available at: https://farmnw.co.uk.
16. Backshall J, Manley J, Rebane M, Nature E (2001) *The Upland Management Handbook* (English Nature) Available at: https://books.google.co.uk/books?id=TUrmAAAACAAJ.
17. FarmIQ (2016) The FeedSmart feed calculator. Available at: https://www.feedsmart.co.nz [Accessed March 15, 2021].
18. Vickers M, Stewart K (2016) Feeding growing and finishing cattle for better returns. *Beef BRP Man 7*. Available at: http://beefandlamb.ahdb.org.uk.
