# Supplementary material for: Fertilization using manure minimizes the trade-offs between biodiversity and forage production in agri-environment scheme grasslands
Source: PLoS One. 2023 Oct 4;18(10):e0290843. doi: 10.1371/journal.pone.0290843 (PMC10550152; doi:10.1371/journal.pone.0290843)
Supplement: Attachment — Submitted filename: Response to reviewers.docx [file pone.0290843.s004.docx]

PONE-D-23-09762

Fertilization using manure minimizes the trade-offs between biodiversity and forage production in agri-environment scheme grasslands

Villa-Galaviz et al.

Comments from the editor

**1. Please ensure that your manuscript meets PLOS ONE's style requirements, including those for file naming. The PLOS ONE style templates can be found at** 
[**https://journals.plos.org/plosone/s/file?id=wjVg/PLOSOne_formatting_sample_main_body.pdf**](https://journals.plos.org/plosone/s/file?id=wjVg/PLOSOne_formatting_sample_main_body.pdf) **and** 
[**https://journals.plos.org/plosone/s/file?id=ba62/PLOSOne_formatting_sample_title_authors_affiliations.pdf**](https://journals.plos.org/plosone/s/file?id=ba62/PLOSOne_formatting_sample_title_authors_affiliations.pdf)

Response: We have reviewed the templates and change the format in the MS.

2. **Thank you for stating the following financial disclosure: 
   "Species ID was funded by Bristol Centre for Agricultural Innovation (BCAI, project no.42). [https://www.bristol.ac.uk/biology/bcai/](https://www.bristol.ac.uk/biology/bcai/)
EVG was funded by a grant from CONACYT, Mexico for graduate studies (381429). [https://conacyt.mx](https://conacyt.mx/)
SMS was supported in part by the UK-SCaPE program delivering National Capability (NE/R016429/1) funded by the Natural Environment Research Council. 
[https://www.ukri.org/councils/nerc/](https://www.ukri.org/councils/nerc/)"**

**Please state what role the funders took in the study.  If the funders had no role, please state: "The funders had no role in study design, data collection and analysis, decision to publish, or preparation of the manuscript." 
If this statement is not correct you must amend it as needed.** 
**Please include this amended Role of Funder statement in your cover letter; we will change the online submission form on your behalf.**

Response: We have removed this information from the manuscript and added the statement to the cover letter.

**3. Thank you for stating the following in the Acknowledgments Section of your manuscript: 
   "We would like to thank Natural England for permission to use their site for fieldwork, especially the managers of Ingleborough National Nature Reserve, C. Newlands and A. Hinde. We are grateful to J. Deeming, M. Pavett R. Barnett, E. Clare and C. Godfray for helping in insect identification, E. Grount for field assistance and J. Martinez-Zavaleta for lab assistance. The original manuscript was improved thanks to comments by T. Timberlake and R. Marrs. The project was partly funded by Bristol Centre for Agricultural Innovation (BCAI). EVG was funded by a grant from CONACYT, Mexico for graduate studies (381429). SMS was supported in part by the UK552 SCaPE program delivering National Capability (NE/R016429/1) funded by the Natural Environment Research Council. The authors declare that there is no conflict of interest."**

**We note that you have provided funding information that is not currently declared in your Funding Statement. However, funding information should not appear in the Acknowledgments section or other areas of your manuscript. We will only publish funding information present in the Funding Statement section of the online submission form. 
Please remove any funding-related text from the manuscript and let us know how you would like to update your Funding Statement. Currently, your Funding Statement reads as follows: 
   "Species ID was funded by Bristol Centre for Agricultural Innovation (BCAI, project no.42). [https://www.bristol.ac.uk/biology/bcai/](https://www.bristol.ac.uk/biology/bcai/)
EVG was funded by a grant from CONACYT, Mexico for graduate studies (381429). [https://conacyt.mx](https://conacyt.mx/)
SMS was supported in part by the UK-SCaPE program delivering National Capability (NE/R016429/1) funded by the Natural Environment Research Council. 
[https://www.ukri.org/councils/nerc/](https://www.ukri.org/councils/nerc/)"**

**Please include your amended statements within your cover letter; we will change the online submission form on your behalf.**

Response: Thank you for your meticulous review. We have updated the statement in the cover letter.

**Reviewer #1:**

There are no comments to address for Reviewer 1.

**Reviewer #2:**

1) The long-term nutrient manipulation experiment was ok, please give the initial soil nutrient before the experiment begin.

Response: We contacted the researchers in charge of the experiment. Professor Richard Bardgett informed us that tracking such information is hard. However, he also told us that the initial soil nutrient before the experiment begin should be quite similar to the adjacent meadow where the management has remained similar to what it was . We have added such information in the manuscript.

2) Please add the organic matter and nutrient contents of farmyard manure.

R: We added information on dry matter and nutrient contents (N, P and K) in farmyard manure in the methods section. Ln 137-139.

3) For some figure, please add different letter of a significant difference for all treatments.

Response: We have indicated in figures 1 and 3 the significant differences among treatments

4) Please add the conclusion.

Response: we have separated the conclusions from discussion by adding the appropriate subheading.

**Reviewer #3**:

1) The limitations of the study immediately followed after discussion section. Please see if should be replaced at the end of the article or may be just before conclusion. However, the conclusion section is also missing. Please see if it is required or not.

Response: Thank you for the comments and for noticing this missing part. We have separated the conclusions from discussion by adding the appropriate subheading.

**Reviewer #4:**

1) The abstract could summarize the results or at least mention which indicators were measured.

Response: we added information of the indicators measured in lines 36-38. We have also summarized the results.

2) In the introduction, it would be necessary to present the different fertilizers that will be used. In the last paragraph the objectives are mentioned and it is not clear what the treatments will be.

Response: We added the fertilizer treatments in lines 108-109

3) Many results are presented in the supplementary material. I think that synthetic figures could be elaborated and included in the work. That might avoid having to present the statistical differences in the text. I think the p-values in the text make reading a bit difficult.

Response: We added a new table with the p values of the randomizations for the indicators when compared individually into the main text. This values were previously reported in the supp. Info. We deleted the p values from the paragraph and refer reader to the table. We also removed those results from the supplementary information. Likewise, we also make it clearer the reading of the results in different sections, to reduce p vales being distracting at reading. In the discussion paragraph that compares indicators between fertilization treatments (452-465) differences between treatments are mentioned. It would be good to indicate the meaning of the differences. For example, instead of saying differences in production were observed between FYM and NPK + FYM, saying NPK + FYM produced slightly (x%) more than FYM.

Response: We have added the percentages in the paragraph. Ln 448-450

4) I think the discussion highlights two main conclusions: 1) that FYM optimizes services and 2) that no fertilization maximizes diversity. The first one has more to do with the objectives (and the methodology) of this work, however, in the end, more emphasis is placed on the second one. I understand that in the end the discussion is on a landscape scale, but it would be good to include a final paragraph with the final conclusions that emerge from this work.

Response: Thank you for the comment we have added a paragraph with the conclusions starting in line 536.

5) Line 77) replace grazing intensity with reducing grazing intensity

Response: Thank you for the observation. We changed the wording, see ln 75.

6) Line 114) compare the performance of four … (without the). Except that tratments are mentioned previously.

Response: Thank you, following previous recommendation we added the fertilizer treatments earlier in the paragraph.

7) Line 137) Check dose of fertilizers. I think there are some problems in the calculations (e.g. N = 12*6 = 72 kg/ha/y)

Response: We have corrected the calculations in the method sections and throughout the manuscript.

Line 144) There were four fertilzar treatments. Or no-fertilizad plot were separated?

Response: Thank you, we have edited the manuscript, so it is clear that there were four fertilizer treatments. Ln 136.

Line 247) Normalization does not necessarily provides values between 0 and 1.

Response: Thank you. We have replace this sentence as follows: “this providing values in the same unit for all indicators while retaining the distributional features of each indicator measured”.

**Reviewer #5:**

1) Add kg to units in abstract.

Response: Thank you. We have added the missing information.

2) Statistical analysis: Please elaborate on the specific R package(s) that were utilized.

Response: Thank you for noting it. We added the packages and now the text reads as follows:

“Analyses used the vegan (40), dtplyr (41), plyr (42), and ggplot2 (43) packages R version 4.1.3 (44)”
